# New Appliances and Things Medical

**Published:** 1907-10-26

**Authors:** 


### NEW APPLIANCES AND THINGS MEDICAL.

[We shall be glad to receive, at our Office, 28 & 29 Southampton Street, Strand, London, W.C., from the manufacturers, specimens of all new preparations and appliances which may be brought out from time to time.]

### HUGON'S ATORA BEEF-SUET.

We have received two kinds of this preparation: the one is refined beef-suet, suitable for frying and many other culinary processes; the other is the so-called shredded suet, and is a refined product in a state of very fine mechanical division. This latter form is useful for cakes, puddings, and many other sweet dishes, for which recipes are given. Suet is one of the most digestible of our fat foods, but its digestibility depends very largely upon the state of mechanical division in which it is presented to the digestive tract, and upon its purity. Atora presents in this respect a very great advantage over ordinary suet in that by merely rubbing between the hands it at once crumbles, and thus lends itself well to the most intimate mixing processes. Both varieties are of good quality, and carefully prepared they may be kept almost indefinitely without going rancid or becoming even tainted, and show in many other ways that they are genuine products. The necessary mechanical manipulations to convert beef fat into good cooking suet are very apt to be neglected in the household, and in Hugon's suet these manipulations have already been performed, and we have an article ready for use. We think the preparation is in every way to be recommended.

